# Diagnostic announcement among children with leukemia

**DOI:** 10.1192/j.eurpsy.2023.787

**Published:** 2023-07-19

**Authors:** S. Dhakouani, M. Karoui, N. Hamrouni, R. Kammoun, F. Ellouze

**Affiliations:** psychiatry, Razi hospital, tunis, Tunisia

## Abstract

**Introduction:**

Announcing a diagnosis of leukemia is a difficult process, especially for a vulnerable population of children.

**Objectives:**

Determine the attitude of caregivers in the announcement of diagnosis of leukemia among children.

**Methods:**

A cross-sectional study was conducted at Aziza Othmana hospital department of haematology in Tunisia between June and July 2021.

We have questioned the mothers about the announcement of the diagnosis of leukemia to their children.

**Results:**

We included 31 children with leukemia, 71% of these children were male. Their average age was 10 years ±4.5 with extremes from 4 to 17 years of age.

The majority of the children (80.6%) were of school age. The three children who were six years old were not able to integrate into a school and fifteen children stopped their studies because of their disease.

Acute lymphoblastic leukemia was the most frequent type of cancer (94%).

Fifty five per cent (55%) of these children were not informed of their disease according to their mothers.

**Conclusions:**

The provision of adapted information, through individualized assessments of each child’s needs, can contribute to the improvement of the child’s experience of the disease.

**Disclosure of Interest:**

None Declared

